# Deviation of the upper, middle and lower thirds of the rectum during irradiation of rectum cancer

**DOI:** 10.2478/raon-2025-0020

**Published:** 2025-06-06

**Authors:** Klemen Salmic, Valerija Zager Marcius, Irena Oblak

**Affiliations:** 1 Institute of Oncology Ljubljana, Department of Radiotherapy, Ljubljana, Slovenia; 2 University of Ljubljana, Faculty of Health Sciences, Department of Medical Imaging and Radiotherapy, Ljubljana, Slovenia; 3 Faculty of Medicine, University of Ljubljana, Ljubljana, Slovenia

**Keywords:** rectal movement, rectal cancer, preoperative radiotherapy, radiotherapy

## Abstract

**Background:**

In patients with rectal cancer, daily fluctuations in rectal volume often lead to large deformations of the target volume that cannot be corrected by adjusting the radiation couch. The main aim of the study was to determine the deviation of all three thirds of the rectum from the reference position and to determine whether these deviations are influenced by the alignments to the bony structures (the sacrum) and the posterior rectal wall.

**Patients and methods:**

The conduct of the study was divided into review of the database, contouring of the anatomic structures on the cone-beam computed tomography (CBCT) images, data collection of the deviations and data output, with the alignment to the bony structures (the sacrum) - and the alignment to the posterior rectal wall performed separately.

**Results:**

Fifty preoperatively irradiated patients were included in the study. The analysis revealed statistically significant differences in terms of alignment to the bony structures for different variations of the rectal thirds in the anterior (+), posterior (+), posterior (−), left (−), right (+) and right (−) directions. With regard to the alignment to the posterior rectal wall, the analysis showed statistically significant differences for different variations of the rectal thirds in directions anterior (+), posterior (+), posterior (−), left (−), right (+), right (−) and bone (+). *(The positive value (+) means that the rectum was larger and the negative value (−) means that the rectum is smaller than in the reference position of the CT simulator images.)*

**Conclusions:**

The position of the rectal thirds changes daily in alignment with the bony structures (the sacrum), and in alignment with the posterior rectal wall.

## Introduction

Approximately 50% of cancer patients require radiotherapy as part of their treatment.^1^ Multidisciplinary treatment of rectal cancer has dramatically improved treatment outcomes and enables long-term survival.^2^ For several years, preoperative radiotherapy (RT) has been the standard treatment for locally advanced rectal cancer.^3^ The adverse effects of pelvic radiation are related to the unavoidable radiation exposure to adjacent healthy tissues or organs at risk (OARs), particularly the bladder and other parts of the bowel.^4^ Many researchers have described the effects of rectal filling fluctuations on the treatment of prostate cancer, but very little is known about the effects of these fluctuations on the treatment of rectal cancer. In prostate radiation, the rectum is an OAR and therefore requires a reduction in radiation volume. When radiating rectal cancer, it is important to ensure that the planned target volume includes the gross tumor volume and the clinical target volume (CTV) along with the rectum and mesorectum.^5^ To ensure that the target is covered with an adequate dose, safety margins are created around the target to account for uncertainties in patient setup and target motion.^6^ Valentini *et al*. state that accurate determination of CTV and OAR is a fundamental step in modern RT.^7^ The target (the rectal tumor) is not visible on the cone-beam computed tomography (CBCT) images, that are part of the on-board imager (OBI).^8^ Image-guided radiotherapy (IGRT) has been defined as any imaging review before, during, or after radiation. Its purpose is to improve or verify the accuracy of patient positioning and thus the accuracy of the radiation treatment.^9^ This level of accuracy allows the use of smaller target volumes and better protection of the OAR.^10^ Within four to five weeks of RT, there are changes in the volume and shape of the bladder and rectum, and, in women, cervical motion, resulting in significant changes in target volumes.^11^ The origin and extent of motion can vary with tumors at different sites in the body. In patients with a tumor in the pelvic region, the position of the target depends on the filling of the bladder and bowel as well as the changing position of the cervix and uterine body. In addition, the target (tumor) usually shrinks or, in very rare cases where the tumor is radioresistant it can increase in size during treatment. For this reason, the actual dose a patient receives may differ from the planned dose due to anatomical changes.^6^ Due to intrafractional and especially interfractional movements of organs in the pelvis, larger safety margins are used to cover the target volume of radiation.^12^ Planning the treatment of rectal cancer with RT is a complex task due to the irregular shapes of the target volume and the proximity of OARs, *e.g*. the bladder, small bowel, sigmoid colon and femoral head.^13^ The aim of this study was to determine the deviation of all three thirds of the rectum from the reference position and to determine whether these deviations are influenced by the alignments to the bony structures (sacrum) and the posterior rectal wall.

## Patients and methods

The clinical protocol used in this study was approved by the Ethics Committee (ERIDEK-0063/2021) and the Clinical Trials Protocol Review Committee (ERID-KSOPKR-0063/2022) at the Institute of Oncology Ljubljana.

This retrospective clinical study included 50 patients. Before the CT simulator and radiation therapy were setup, all patients were informed about the hydration protocol and bladder preparation. Patients emptied their bladder and consumed 0.5 liters of water 45 minutes prior to radiation and an oral dose of the chemotherapy tablet capecitabine one hour prior to treatment. Imaging was performed using a CT simulator (Somatom Definition AS CT simulator – Siemens, Erlangen, Germany) covering the region from the L2-L3 junction to 5 cm below the initial mark at the beginning of the anus. Both the CT simulator and the CBCT images had a scan thickness of 3 mm. The linear accelerator used in this study was a TrueBeam (Varian Medical Systems, Palo Alto, USA). Anatomical contouring was performed using ECLIPSE software (Varian), delineating the rectum, sacrum and bladder on the transverse CBCT images, with the rectum fully delineated on the reference CT image. Automatic fusion of the daily CBCT and planning CT images facilitated alignment to the sacrum and posterior rectal wall, with data collected for each rectal segment.

Alignment with the bony structures, in particular the sacrum, was performed in all six directions (three translational and three rotational directions), with measuring points defined for the upper, middle and lower rectal segments. The measurement points for the upper rectum were: the beginning of the sacroiliac joint in the caudal-cranial direction; for the lower rectum: the end of the sacrum/coccyx; and for the middle rectum: the midpoint between the two predefined points – the femoral head to the transition into the femoral neck. In 20% of the cases, where the predefined measurement points were not accessible, simulated points were used. In these cases, the following points were simulated: the beginning of the rectum in the cranial-caudal direction for the upper rectum, the beginning of the femoral neck for the middle rectum and the measured difference between the two points mentioned above for the lower rectum. In this case, a fixed point in the lower rectum was determined and after fusion with the posterior rectal wall, the deviation was measured, which represents the deviation of the bony structures, *i.e*. the sacrum.

**FIGURE 1.**
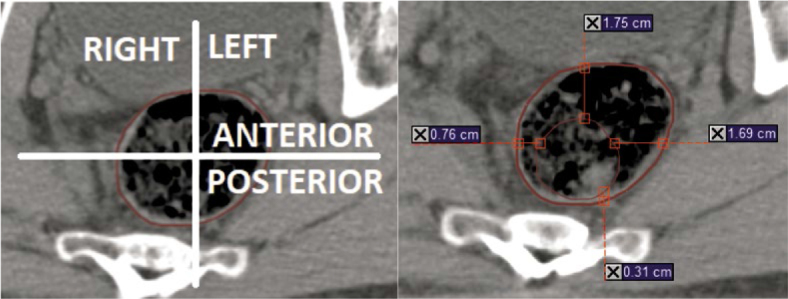
Direction of measurements taken and example of measurements used in the study. The bold brown line is the reference position on CT image; the thin brown line is the daily position on cone-beam computed tomography (CBCT)

Alignment to the posterior rectal wall was performed manually in the anterior-posterior direction on the sagittal scans, with deviations measured at defined points. The measurement of deviations from the bone structure was limited to the anterior-posterior direction to maintain anatomical integrity. Due to the inclusion of regional lymph nodes in the treatment field, the automatic alignment of the rectum was omitted in favor of the alignment of the bone structure. Measurements included anterior, posterior, left and right deviation of the rectal wall on transverse scans on reference CT images (Figure 1). The caliper was always used perpendicular to a rectal wall. We favored the positive value because we focused more on the possible location of the target from the treatment volumes. Superior and inferior directions were not measured due to the predefined measurement points, as it would be difficult to determine the exact height of the rectal thirds in the other views of the scan. When measuring deviations, the values were measured as a positive value (+) if the rectal wall deviated outside the reference position and as a negative value (-) if the rectal wall was inside the reference position in CT simulator scans. The software programs Microsoft Excel 2010 and IBM SPSS Statistics 26 were used to analyze and evaluate the collected data. The Shapiro-Wilk test was used to determine the normality of the sampling distribution. Based on the results, the parametric (ANOVA, T-test, Mann-Whitney test) or non-parametric tests (Wilcoxon test, Kruskal-Willis test) were then applied. The Spearman correlation was also used when carrying out various correlations. A statistically significant difference was assessed at p-value p ≤ 0.05 (a 5% level of risk).

## Results

Fifty patients were included in the study, 30 men (60%) and 20 women (40%). The average age of the included patients was 60.5 years (37–81). Three hundred and fifteen CBCT images were reviewed and analyzed (average 6.3 per patient). We analyzed the difference between the two types of alignment (bony structures, *i.e*., sacrum/posterior rectal wall). The following dependent variables were analyzed: anterior (+), anterior (−), posterior (+), posterior (−), left (+), left (−), right (+), right (−), bone (+) in bone (−). A positive value (+) means that the position of the rectal wall was larger and outside the reference position, while a negative value (−) means that the position of the rectal wall was smaller and inside the reference position.

Regarding the alignment with the bone structures, the analysis revealed statistically significant differences for the following variables in anterior (+) direction between lower and upper third (p = 0.001) and between lower and middle third (p = 0.002), in posterior (+) direction between lower and middle third (p = 0.003), in posterior (−) direction between middle and upper third (p = 0.029) and between lower and upper third (p = 0.040), in the left (−) direction between lower and upper third (p = 0.004) and between lower and middle third (p = 0.024), in the right (+) direction between lower and upper third (p = 0.003), in the right (−) direction between lower and upper third (p = 0.014) and between lower and middle third (p = 0.001). No statistically significant differences were found for other dependent variables. Deviations relative to the bone structures are not shown, i.e. there were no deviations because the alignment was performed relative to the bone structures.

**FIGURE 2.**
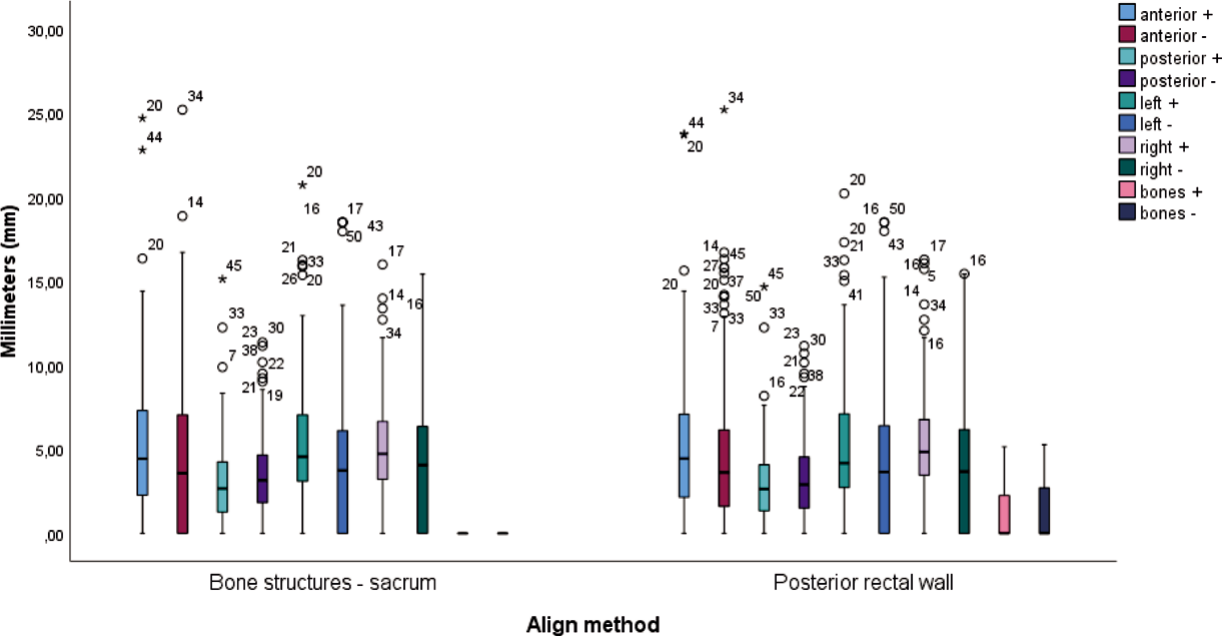
Rectal thirds when using a different alignment method.

With regard to alignment with the posterior rectal wall, the analysis revealed statistically significant differences for the following variables in the anterior (+) direction between lower and upper thirds (p = 0.01), in the anterior (+) direction between lower and middle thirds (p = 0.003), in the posterior (+) direction between lower and upper thirds (p = 0.035) and between lower and middle thirds (p = 0.001), in the posterior (−) direction between middle and upper thirds (p = 0.010) and between lower and upper thirds (p = 0.028), in the left (−) direction between lower and upper thirds (p = 0.008) and between lower and middle thirds (p = 0.021), in the right (+) direction between lower and upper thirds (p = 0.008), in the right (−) direction between lower and upper thirds (p = 0.010) and between lower and middle thirds (p = 0.0001), in the bone (+) direction between lower and upper thirds (p = 0.002) and between lower and middle thirds (p = 0.004). No statistically significant differences were found for the other dependent variables. Figure 2 shows the rectal thirds for different alignment methods.

### Correlations of the different alignment methods with gender

**FIGURE 3.**
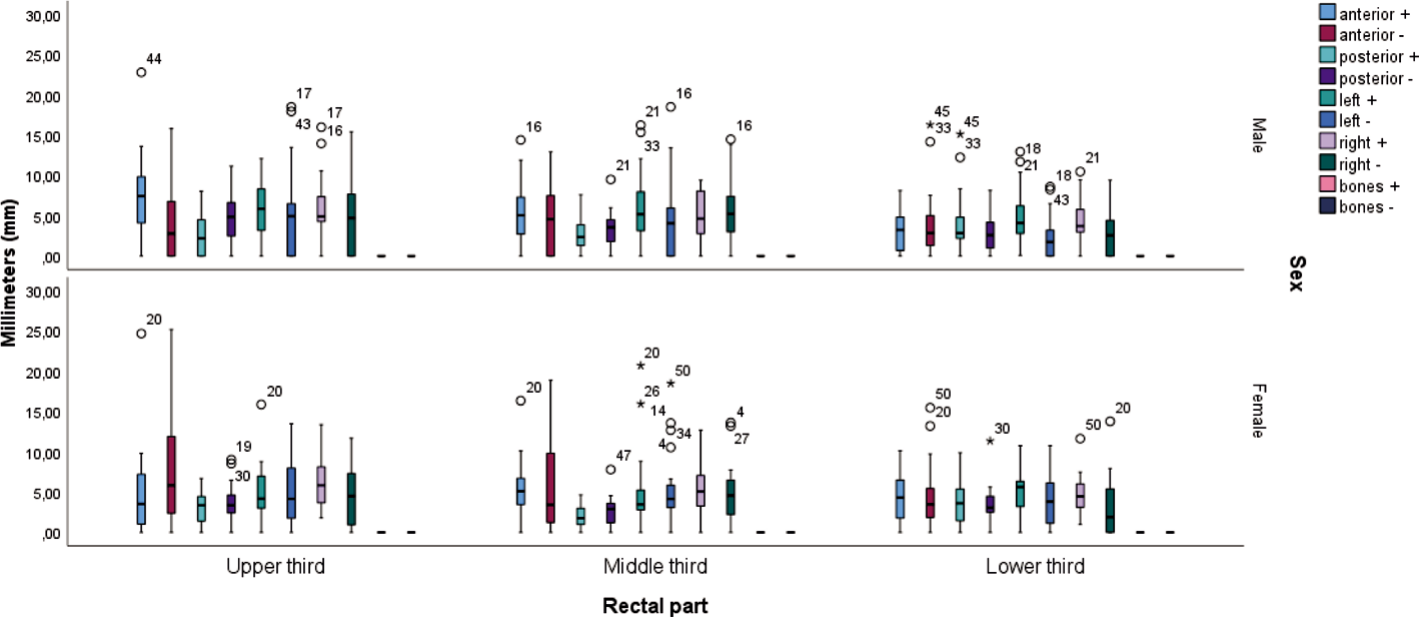
Rectal thirds when using a method to align the bone structures – the sacrum compared to gender.

**FIGURE 4.**
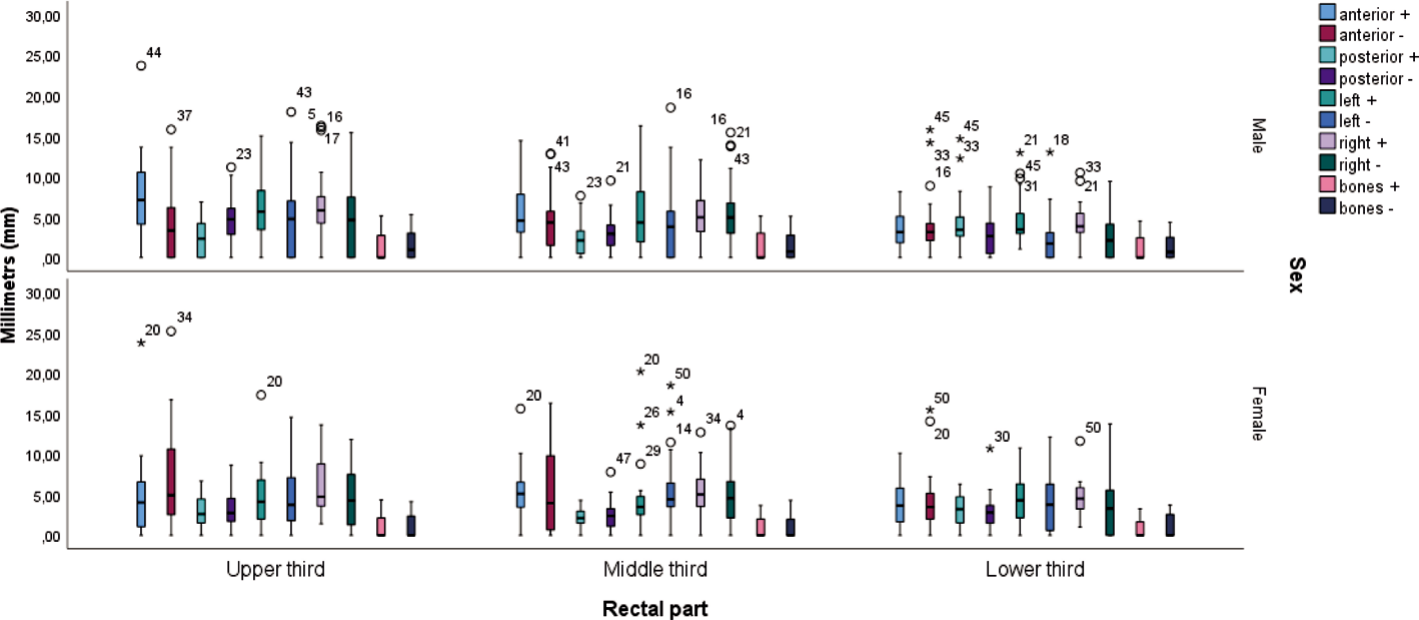
Rectal thirds when using a method to align the posterior rectal wall compared to gender.

Figures 3 and 4 show the rectal thirds with different alignment methods as a function of gender. When targeting the bony structures, the differences between male and female gender were statistically significant for the variable (upper third) anterior (+) (p = 0.020) and the variable (lower third) left (−) (p = 0.032), while the differences for the other dependent variables were not statistically significant. When targeting the posterior rectal wall, the differences between the male and female gender were statistically significant for the variable (upper third) anterior (+) (p = 0.022), the variable (lower third) left (−) (p = 0.031) and the variable (upper third) posterior (+) (p = 0.035), while the differences for the other dependent variables were not statistically significant. The mean value (Mv) of the variable (upper third) anterior (+) was higher in male **(Mv = 29.33 mm)**, while it was lower in females **(Mv = 19.75 mm)**. The Mv of the variable (upper third) posterior (+) was lower in males **(Mv = 23.87 mm)**, while it was higher in females **(Mv = 27.95 mm)**. The Mv of the variable (lower third) left (−) was higher in females **(Mv = 30.83 mm)**, while it was lower in males **(Mv = 21.95 mm)**.

### The volume of the rectum and bladder

**FIGURE 5.**
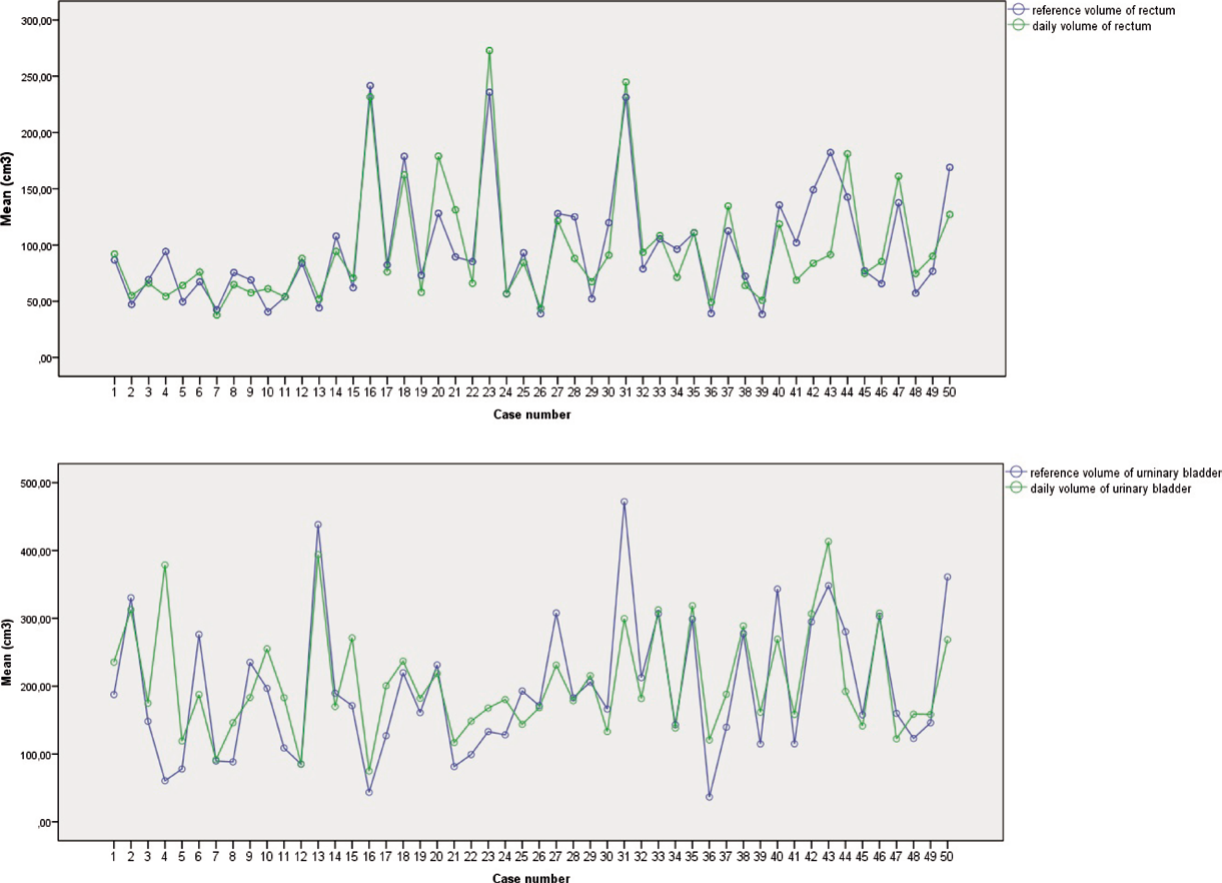
Fluctuations in the daily and reference volume of the rectum and urinary bladder.

Rectal and bladder volumes were analyzed by subtracting the daily volume (DVo) from the reference volume (RVo) and then statistically examining the differences. The average difference between RVo and DVo for the rectum was –1.98 cm^3^, which means that the DVo of the rectum was on average 2 cm^3^ smaller than the RVo. The largest difference between RVo and DVo was –90.71 cm^3^ while the smallest difference was 50.77 cm^3^. The average difference between RVo and DVo for the bladder was –10.42 cm^3^, which means that the DVo of the bladder was on average 10.42 cm^3^ smaller than the RVo. The largest difference between RVo and DVo was –318.20 cm^3^, while the smallest difference was 172.52 cm^3^. The differences between the DVo and RVo of the rectum and bladder were not statistically significant. The fluctuations in the daily and reference volumes of the rectum and bladder are shown in Figure 5.

## Discussion

The aim of this study was to determine the deviation of all three thirds of the rectum from the reference position and to determine whether these deviations are influenced by the alignments to the bony structures, *i.e*. the sacrum and the posterior rectal wall. The reference position was the position of the rectum obtained from a CT scan when the patient was being prepared for radiation. The study included fifty patients with a review and analysis of 315 CBCT images. The analysis focused on differences in alignment methods in relation to bone structures and the posterior rectal wall in the upper, middle and lower thirds of the rectum. Many researchers have described the effects of fluctuations in rectal filling on the treatment of prostate and uterine cancer, but very little is known about the effects of these fluctuations on the treatment of rectal cancer.^5,14^ Haken *et al*. reported that the prostate and seminal vesicles were capable of an average movement of 1 cm in response to filling of the rectum with contrast at the time of simulation.^15^ Interfraction deviation was evaluated for movement of the prostate and seminal vesicles for cases in which either the bladder or rectum was fullest.^16–17^ Mak *et al*. found that anteroposterior motion of the seminal vesicle correlated with posterior point motion of the bladder and rectum.^16^ Nuyttens *et al*. have described the extent of motion of the CTV for conventional adjuvant therapy of the rectum, perirectal tissue, and regional lymph nodes, but not that of the organs themselves.^18^ Brierley *et al*. sought to evaluate the difference in movement of the upper, middle, and lower thirds of the rectum and the effect of changes in bladder volume. To assess the movement of the mesorectum, they used an intrarectal contrast agent. They found that the greatest movement of the rectum occurred in the upper third^19^, which is consistent with our results. A similar study was performed by Alickikus *et al*., who showed that the mesorectum is a structure that exhibits internal movement due to its location and the physiologic movement of adjacent organs such as the rectum and bladder.^20^ In our study, rectal tumor interfraction displacement was our research interest and analysis.

Statistically significant differences in alignment with bony structures were found in several variables, especially in the lower third compared to the upper third and the middle third of the rectum. These differences were observed in variables such as anterior (+), posterior (+), left (−) and right (+). However, no differences were observed in relation to the bone structures. Statistically significant differences were also observed between the different variables for alignment with the posterior rectal wall, especially between the lower and upper third. Statistically significant differences were observed for anterior (+), posterior (+), left (−), right (+) and bone (+). In conclusion, the above dependent variables indicate different variations in different sections of the rectum, further highlighting the variations in the rectal wall compared to a single section.

Based on our results, we believe that the safety margins for the different parts of the rectum should not be uniform but should be reviewed and adjusted in case of major variations. The larger deviation was found in the upper third of the rectum. Therefore, we should be more cautious in patients with a rectal tumor in the upper third of the rectum and check the target volume daily by CBCT.

Regarding the influence of gender on the different alignment methods, statistically significant differences were found for certain variables such as anterior (+) and left (−), especially in the upper third for alignment to both the bony structures and the posterior rectal wall. These results indicate a correlation between the different anatomy of the sexes and the alignment of the rectal wall. From the results described above, it can be summarized that on average there are greater deviations in the female gender, which was also confirmed by Nijkamp *et al*. who found in his study that greater safety margins are required in women.^11^ We assume that the gender-specific differences are mainly a consequence of the peristaltic process and the body anatomy itself. In men, the organ that influences the movement of the rectum is the bladder, while in women the uterus and cervix play an important role.

In addition, the study analyzed the volume difference between the reference volume and the daily volume for both the rectum and the bladder. It can be observed that the largest and smallest fluctuations are considerable in both the rectum and the bladder, which illustrates the changing volumes during the daily radiation. On average, the daily volume of the rectum was about 2 cm^3^ smaller than the reference volume. Similarly, the daily volume of the bladder was about 10.42 cm^3^ smaller than the reference volume. However, these differences were not statistically significant. In our study bladder variations were checked with CBCT and ultrasound before treatment. In case of larger deviations, we ask the patient to empty the bladder or drink a little more water. We also contour larger internal target volume (ITV) in the anterior direction in the area of the bladder for 0.5–1 cm.

One limitation of our study is the relatively small number of patients included, although the number of measurements was large. Another limitation is possible errors in the delineation of anatomical structures related to the lower quality of CBCT images and the presence of artifacts. Some CBCT scans were excluded due to the above-mentioned problems, which limits the accuracy of the results. On average, we obtained 6 CBCT scans out of the planned 22 fractions during the treatment process. If the CBCT scans were performed daily, they would provide a more detailed and accurate view of the movement of the rectal wall. The presence of gas and stool in the rectum interfered with the deformations, which made the measurements difficult. We also encountered certain challenges in performing the measurements while assessing the deviations. We must also keep in mind that only three patients with tumors located in the upper third were included, which is not a representative sample in terms of tumor location. In addition, delineation in the upper rectal area was extremely difficult due to the different filling of the rectum, as well as the rectosigmoidal border, which is sometimes less visible or more difficult to define due to the curvature of the bowel.

In conclusion, this study provides a valuable insight into the alignment of the rectal wall using CBCT imaging and shows statistically significant differences between the different thirds and possible correlations with gender. The position of the rectal thirds changes daily, both in alignment with the bony structures (the sacrum), and in alignment with the posterior rectal wall. The greatest deviation has been observed in the upper third of the rectum, so we need to be particularly careful in these patients and should perform a daily target volume assessment. In addition, analyzing the volume differences allows for a better understanding of rectal and bladder-related dynamics.
